# Depression and anxiety among people living with and beyond cancer: a growing clinical and research priority

**DOI:** 10.1186/s12885-019-6181-4

**Published:** 2019-10-11

**Authors:** Claire L. Niedzwiedz, Lee Knifton, Kathryn A. Robb, Srinivasa Vittal Katikireddi, Daniel J. Smith

**Affiliations:** 1 Institute of Health & Wellbeing, University of Glasgow, Glasgow, Scotland, UK; 2 University of Strathclyde, Centre for Health Policy, Glasgow, Scotland, UK; 3 Mental Health Foundation, Glasgow, Scotland, UK; 4 MRC/CSO Social and Public Health Sciences Unit, University of Glasgow, Glasgow, Scotland, UK

**Keywords:** Mental health, Psychiatry, Cancer, Multimorbidity, Depression, Anxiety, Oncology, Survivorship

## Abstract

**Background:**

A cancer diagnosis can have a substantial impact on mental health and wellbeing. Depression and anxiety may hinder cancer treatment and recovery, as well as quality of life and survival. We argue that more research is needed to prevent and treat co-morbid depression and anxiety among people with cancer and that it requires greater clinical priority. For background and to support our argument, we synthesise existing systematic reviews relating to cancer and common mental disorders, focusing on depression and anxiety.

We searched several electronic databases for relevant reviews on cancer, depression and anxiety from 2012 to 2019. Several areas are covered: factors that may contribute to the development of common mental disorders among people with cancer; the prevalence of depression and anxiety; and potential care and treatment options. We also make several recommendations for future research. Numerous individual, psychological, social and contextual factors potentially contribute to the development of depression and anxiety among people with cancer, as well as characteristics related to the cancer and treatment received. Compared to the general population, the prevalence of depression and anxiety is often found to be higher among people with cancer, but estimates vary due to several factors, such as the treatment setting, type of cancer and time since diagnosis. Overall, there are a lack of high-quality studies into the mental health of people with cancer following treatment and among long-term survivors, particularly for the less prevalent cancer types and younger people. Studies that focus on prevention are minimal and research covering low- and middle-income populations is limited.

**Conclusion:**

Research is urgently needed into the possible impacts of long-term and late effects of cancer treatment on mental health and how these may be prevented, as increasing numbers of people live with and beyond cancer.

## Background

A cancer diagnosis can have a wide-ranging impact on mental health and the prevalence of depression and anxiety among people with cancer is high [1, 2]. Among those with no previous psychiatric history, a diagnosis of cancer is associated with heightened risk of common mental disorders, which may adversely affect cancer treatment and recovery, as well as quality of life and survival [3]. People who have previously used psychiatric services may be particularly vulnerable and at greater risk of mortality following a cancer diagnosis [4]. However, the mental health needs of people with cancer, with or without a prior psychiatric history, are often given little attention during and after cancer treatment, which is primarily focused on monitoring physical health symptoms and side effects. Advances in the earlier detection of cancer and improved cancer treatments means that people are now living longer with cancer, presenting a significant global challenge. The total number of people who are alive within 5 years of a cancer diagnosis was estimated to be 43.8 million in 2018 for 36 cancers across 185 countries [5], and in the United States alone, the number of cancer survivors is projected to rise exponentially from 15.5 million in 2016 to 26.1 million in 2040 [6].

The main objective of this article is to argue that more research is needed into the prevention, care and treatment of co-morbid depression and anxiety among people with cancer and highlight it as a growing clinical and policy priority. For background and to support our argument, we provide a current evidence review of systematic reviews relating to common mental disorders amongst people living with and beyond cancer. We cover the factors that may increase the risk of experiencing co-morbid depression and anxiety, epidemiology, and potential care and treatment options.

We searched three key electronic databases: Medline, PsycINFO and CINAHL (Cumulative Index to Nursing and Allied Health Literature) for relevant reviews (favouring those using systematic methods) using the following search terms: (neoplasm OR carcinoma OR tumo*r OR cancer) AND (depression OR anxiety) AND review. Only English language articles were considered and searches were limited to the years 2012 to 2017 and updated during February 2019. These years were considered adequate to capture the main themes relating to cancer and common mental disorders in the current literature. The references of highly relevant articles were scrutinised for additional papers and a Google search for important grey literature was also conducted. A minority of significant research articles known to the authors were also consulted.

## Main text

### Factors influencing the development of depression and anxiety among people with cancer

**Fig. 1:**
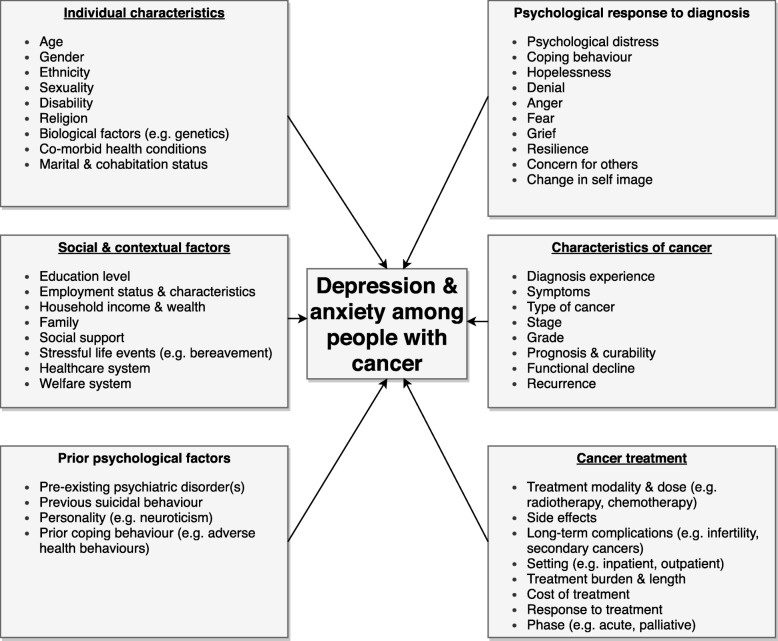
Factors that may contribute to depression and anxiety among people living with and beyond cancer

A variety of factors are likely to interact to influence the development of depression and anxiety among people with cancer (summarised in Fig. 1), but these are not well understood [1], and require further research. Individual risk factors that may increase the risk of depression, similar to the general population, include demographic factors, such as age and gender, and social and economic factors such as unemployment, fewer educational qualifications and a lack of social support [7]. The development of depression and anxiety among people with cancer is also likely to depend on factors at the structural level, including healthcare costs and access, as well as access to welfare support, such as disability benefits, as cancer can have a significant financial impact [8, 9]. Several psychological factors are also important. A key factor is the presence of pre-existing mental health problems and their severity. Research has demonstrated that individuals who have previously accessed mental health services before a cancer diagnosis experience excess mortality due to certain cancers, which may reflect late diagnosis, inadequate treatment and a higher rate of adverse health behaviours [4, 10]. Personality factors, such as neuroticism, and existing coping skills may also contribute [11]. The risk of suicide among people with cancer is higher than the general population for certain diagnoses that tend to have poorer prognoses, such as mesothelioma and lung cancer, especially in the first 6 months after diagnosis [12, 13]. Individuals who have previously engaged in suicidal behaviour are likely to be particularly vulnerable.

The individual psychological response to a cancer diagnosis is also likely to be an important component. The experience of being diagnosed, particularly if the diagnosis has been delayed, can be a significant source of distress and can impact on illness acceptance [14]. Feelings of hopelessness, loss of control and uncertainty around survival and death can also have a detrimental impact, particularly in patients with a poor prognosis. Anxiety around a cancer diagnosis can also lead to sleep disturbance, which may increase the risk of depression [15]. The stigma surrounding both mental illness and certain types of cancer, such as lung cancer, can lead to feelings of guilt and shame, which could contribute to the onset of depression. For example, the link between smoking and lung cancer can lead to some patients blaming themselves for their illness and experiencing stigma if they have engaged in smoking [14].

A variety of factors related to the cancer and its treatment are likely to impact on the development of depression and anxiety, including the type of cancer, stage and prognosis. Cancer treatments including immunotherapy and chemotherapy may induce depression through particular biological mechanisms, such as inflammatory pathways, and some medications used to treat chemotherapy-induced nausea can reduce dopaminergic transmission, which is implicated in the development of depressive symptoms [16]. The use of steroids in cancer treatment can induce depression [17], and androgen deprivation therapy in the treatment of prostate cancer is also associated with increased risk [18]. The physical symptoms of specific cancers can also contribute to depression (e.g. incontinence and sexual dysfunction associated with prostate cancer) [19]. Iatrogenic distress is also commonly reported amongst patients, which could increase the risk of experiencing later problems with depression and anxiety, including post-traumatic stress disorder [20]. This is often related to a combination of poor communication, a lack of consideration of psychological concerns and disjointed care [14, 20].

### Prevalence of depression and anxiety among people with cancer

The prevalence of common mental disorders among people with cancer varies widely in the published literature. The mean prevalence of depression using diagnostic interviews is around 13% and using all assessment methods it varies from approximately 4 to 49% [2, 21]. This wide variation is due to several factors including the treatment setting, type of cancer included and method used to screen for symptoms (e.g. interview by trained psychiatrist or self-report instrument). The estimated prevalence of depression was found to be 3% in patients with lung cancer, compared to 31% in patients with cancer of the digestive tract, when diagnostic interviews were used [21]. A meta-analyses of 15 studies meeting a number of quality criteria, including the use of diagnostic interviews, found that the estimated prevalence of depression varied across treatment settings (5 to 16% in outpatients, 4 to 14% in inpatients, 4 to 11% in mixed outpatient and inpatient samples, and 7 to 49% in palliative care) [2]. There is no universal standardised tool which is recommended for depression screening in patients with cancer and the method used is likely to differ depending on the treatment setting. A meta-analysis of screening and case finding tools for depression in cancer settings identified 63 studies that used 19 different screening tools for depression [22]. Common screening methods for depression include semi-structured diagnostic interviews, the Hospital Anxiety and Depression Scale - depression subscale (HADS-D) and Center for Epidemiologic Studies Depression Scale (CESD), which are designed to measure the severity of depressive symptoms.

An important aspect that needs to be considered is the timing of increased psychiatric risk. Studies demonstrate that depression tends to be highest during the acute phase and decreases following treatment, but again this likely differs depending on the type of cancer and prognosis [21]. Using diagnostic interviews, the prevalence of depression during treatment was found to be 14%, 9% in the first year after diagnosis and 8% a year or more after treatment in a meta-analysis of 211 studies [21]. Of the 238 cohorts included, around 30% included only breast cancer patients and there is a need for research including rarer types of cancer.

As well as the type of cancer, the type of mental health outcome considered is also important and fewer studies have examined anxiety. A systematic review and meta-analysis study focusing on patients with ovarian cancer found that anxiety tended to be higher following treatment (27%) and during treatment (26%), and was lowest pre-treatment (19%) [23]. The heightened anxiety observed post-treatment may be due to reduced clinical consultations and support following treatment, potential transfer to a palliative setting, and fear of recurrence. Fear of recurrence is one of the most commonly reported issues and an important area of unmet need for cancer survivors [24]. A lack of outward physical symptoms in ovarian cancer also means that self-monitoring is difficult [23]. In the same study of ovarian cancer patients, depression was highest before treatment (25%) and during treatment (23%), and reduced following treatment (13%). This is in the context of a lifetime prevalence for clinical depression and anxiety of around 10 and 8%, respectively, amongst women in the UK [23, 25].

A similar systematic review of depression and anxiety among patients with prostate cancer found that anxiety tended to be highest pre-treatment (27%) and lowered during treatment (15%) and post-treatment (18%) [26]. Rates of depression were relatively similar following treatment (18%), during treatment (15%) and pre-treatment (17%), with the 95% confidence intervals for these estimates largely overlapping. For reference, the prevalence of clinical depression and anxiety in men aged over 65 years is less than 9 and 6%, respectively [26]. A systematic review on the prevalence of psychological distress among testicular cancer survivors demonstrated that around one in five experienced clinically significant anxiety, compared to one in eight among general population controls, with fear of recurrence again being one of the key issues reported [27]. However, depression was no more prevalent amongst those surviving testicular cancer compared to the general population. In Scotland, the prevalence of depression was found to be highest in patients with lung cancer (13%), followed by gynaecological cancer (11%), breast cancer (9%), colorectal cancer (7%), and genitourinary cancer (6%) [28]. The authors found depression to be more likely among younger and more socially disadvantaged individuals. In addition, 73% of the patients with depression were not receiving treatment for their mental health. Further research is needed to ascertain the factors which contribute to the uptake and efficacy of treatment for depression. This study also only considered people with cancer who had attended specialist cancer clinics within a defined time period, which likely excluded people who were diagnosed many years ago.

The longer-term psychological impact of cancer has received comparatively little research. The few studies in this area have mainly focused on women with breast cancer and demonstrate that depressive symptoms can persist for over 5 years after diagnosis, though the prevalence of anxiety was not elevated compared to the general population [29]. A systematic review of the prevalence of depression and anxiety among long-term cancer survivors, including all types, found that anxiety was more prevalent among cancer survivors, compared to healthy controls [30]. Few studies have focused specifically on younger cancer survivors and more research is needed in this area. A representative study of young adult cancer survivors aged 15 to 39 years in the United States demonstrated that moderate (23% vs 17%) and severe (8% vs 3%) mental distress were significantly higher in those living with cancer for at least 5 years after diagnosis, compared to controls [31]. 75 and 52% of people with cancer with moderate and severe distress, respectively, had not talked to a mental health professional, with the cost of treatment a potential barrier. Limitations of this study included the focus on self-reported mental distress and not clinical depression or anxiety, as well as the relatively small sample size.

Many studies in this area have a poor response rate, lack representativeness, are based on a small sample of patients (often with the most common types of cancer), which often exclude those with cognitive impairment and patients who are too physically or mentally unwell to take part [32]. Future studies would benefit from using administrative health data [33], for example, linking together cancer registries, inpatient and outpatient records and prescribing data. There are also a lack of studies covering populations from low- and middle-income countries [34]. The estimated prevalence of comorbid common mental disorders is likely to vary depending on the country studied, due to factors such as the health and welfare system. These factors may influence mental health inequalities among people with cancer, which has received little research focus. In a Scottish study, depression was found to be higher in the least advantaged groups (19%), compared to the most advantaged (10%) [35]. Cancer and comorbid anxiety was also unequally distributed; in the least advantaged groups around 12% had both conditions, compared to 7% among the most advantaged [35]. Further research is needed in this area to quantify, monitor and prevent inequalities among people with cancer.

It should also be highlighted that the psychological impact of cancer may not always be negative and many people will not experience problems with depression and anxiety. Experiencing temporary distress related to a cancer diagnosis may lead to positive psychological changes in the long-term whereby individuals feel a greater appreciation of life and are able to re-evaluate their priorities [36]. The factors that protect against the development of common mental disorders and contribute to positive mental health among people living with and beyond cancer merits further research.

### Treatment and management of depression and anxiety among people with cancer

To effectively manage and treat depression and anxiety among people with cancer, symptoms must first be identified. However, several social and clinical barriers have been reported. A key issue is the lack of physician time for assessing symptoms. There can also be a normalisation of distress and attribution of the somatic symptoms of depression and anxiety to the cancer. Patients may not disclose psychiatric symptoms because of the stigma surrounding mental health conditions [37]. Screening for depression and anxiety among patients with cancer is also only of value if it leads to effective treatment and support that is able to improve patient outcomes. Patients may be more reluctant to discuss their mental health needs if they perceive a lack of effective treatment options.

The existing evidence for treating anxiety and depression among patients with cancer is limited and of varying quality [38]. Studies with small sample sizes are common; this mitigates against the detection of meaningful changes in patient outcomes and these studies often suffer from a high rate of attrition, which likely reflects the high symptom burden and reduced survival in this patient population [39]. Systematic reviews demonstrate there is a preponderance of studies from the United States, which include a high number of studies focusing on female patients with breast cancer [40]. However, these studies demonstrate that psychotherapy, psychoeducation and relaxation training may have small to medium short-term effects on relieving emotional distress and reducing symptoms of anxiety and depression, as well as improving health-related quality of life. The evidence for pharmacological treatment of depression with antidepressants is mixed - there are very few studies in this area and those that exist are of low quality [41]. There is also concern around potential side effects of antidepressants and drug interactions that may affect the efficacy of cancer treatments [42].

A systematic review and meta-analysis focusing on cognitive behavioural therapy (CBT) found that it may be effective in reducing depression and anxiety and improving quality of life in patients with cancer in the short-term, but potential long-term effects were only sustained for quality of life [43]. However, in this meta-analysis the included participants were primarily women with breast cancer and there are a lack of studies covering other cancer types. It is likely that collaborative care interventions which involve partnership between psychiatry, clinical psychology and primary care, overseen by a care manager are likely to be most effective in the management and treatment of depression amongst people with cancer [44]. Treatment should be based on patient preference and also take into account potential adverse side effects [44]. In a UK-based study it was found that only a third of patients with cancer and related psychological or emotional distress were willing to be referred for support [45]. Qualitative studies also demonstrate that patients often do not want to discuss their feelings with nurses during cancer treatment [46]. However, patients valued having the option to talk about their emotions, but they preferred to choose with whom and when. There is therefore a need for further research into some of the barriers to obtaining mental health support among those affected by cancer and experiencing distress to prevent future problems.

The self-management of psychological distress among people with cancer may be beneficial and could help prevent distress becoming clinical depression or anxiety. Self-management can be defined as: *“The individual’s ability to manage the symptoms, treatment, physical and psychosocial consequences and lifestyle changes inherent in living with a chronic condition. Efficacious self-management encompasses the ability to monitor one’s condition and to affect the cognitive, behavioural and emotional responses necessary to maintain a satisfactory quality of life. Thus, a dynamic and continuous process of self-regulation is established.”* [47]*.* Studies on self-management, cancer and psychological distress have focused on the treatment phase, with fewer investigating interventions following treatment or at the end of life [48]. There is evidence to suggest that self-management of psychological distress in cancer can help to empower patients and families to care for themselves in a way which is preferable for them. Self-management interventions that have shown promise include education, monitoring, teaching and counselling to help patients manage the short- and long-term physical and psychosocial effects of cancer [48]. However, a recent systematic review examining the impact of self-management interventions on outcomes including quality of life, self-efficacy and symptom management (such as psychological distress) amongst cancer survivors demonstrated a lack of evidence to support any specific intervention and found that the six included interventions lacked sustainability, bringing into question their long-term effectiveness and value for money [49]. Again, the included studies were dominated by women with breast cancer, with only two covering other cancers.

Effective treatment and management strategies may also differ according to the demographic group affected. In a report by CLIC (Cancer and Leukaemia in Childhood) Sargent which surveyed 146 young people with cancer, keeping in touch with friends and family, talking to others with similar experiences and access to the internet in hospital were reported to help maintain mental health during cancer treatment [50]. Of the young people who mentioned they would find it helpful to talk to other people with similar experiences, 60% said they would prefer to do this online. Young people also reported that the available services were not tailored to deal with those aged under 18 or the emotional impact of cancer. In addition, those who accessed services mentioned that there is a lack of suitable long-term emotional support. Just over 40% of the young people who took part did not access support for their mental health needs.

It is clear that a more personalised approach to supporting the psychological health of people with cancer is needed [51]. Some people may not want or require support or treatment, others will be able to self-manage, and some may have more complex needs that require more intensive follow-up and support. At diagnosis, the psychological health of patients should be considered alongside their physical health and sources of support offered. Needs and symptoms may also change over time. Evaluation of more recent personalised approaches to follow-up care that have been adopted in several areas including England and Northern Ireland [51] are needed to understand the role they may have in preventing longer term depression and anxiety amongst cancer survivors.

A key barrier affecting research progress in this area is funding [52]. In the UK, money spent on research into the biology of cancer was more than five times than that spent on ‘Cancer Control, Survivorship and Outcomes’ during 2017/18 [53]. Research into the mental health and wellbeing of people living with and beyond cancer is likely to only be a small part of this. Research is urgently needed in this area as more people survive cancer and for some cancers, such as multiple myeloma and colorectal cancer, risk is increasing in younger cohorts [54]. The long-term (those that begin during treatment and continue afterwards) and late effects of cancer treatment (those that begin after treatment is completed), such as secondary cancers, infertility, chronic pain and insomnia, are likely to affect the mental wellbeing of cancer survivors, potentially contributing to depression and anxiety [6]. The National Cancer Research Institute (NCRI) in the UK have also recently highlighted research into the short-term and long-term psychological impacts of cancer and its treatment as a key priority, following surveys of over 3500 patients, carers, and health and social care professionals [55].

## Conclusion

The mental health of people living with and beyond cancer in its various types and stages is an important and growing research and clinical priority. Compared to the general population, the prevalence of anxiety and depression is often higher among people with cancer, but estimates vary due to a number of factors, such as the type and stage of cancer. Patients often do not obtain psychological support or treatment. This is likely due to several factors, including lack of awareness and identification of psychiatric symptoms, an absence of support available or offered, lack of evidence around effective treatments, stigma, and patient preference. In particular, we highlight the lack of high-quality research into the mental health of long-term cancer survivors, the potential impact of long-term and late effects of cancer treatment, and the few studies focused on prevention. Further research that includes the less common types of cancer is required, as well as the inclusion of younger people and populations from low- and middle-income countries. Given the increasing numbers of people living with and beyond cancer, this research is of timely importance.

## Data Availability

All data generated or analysed during this study are included in this published article.

## Abbreviations

CBT: Cognitive behavioural therapy
CESD: Center for Epidemiologic Studies Depression Scale
CINAHL: Cumulative Index to Nursing and Allied Health Literature
CLIC: Cancer and Leukaemia in Childhood
HADS-D: Hospital Anxiety and Depression Scale - depression subscale
NCRI: National Cancer Research Institute
